# The predictive value of resting heart rate in identifying undiagnosed diabetes in Korean adults: Korea National Health and Nutrition Examination Survey

**DOI:** 10.4178/epih.e2022009

**Published:** 2022-01-03

**Authors:** Dong-Hyuk Park, Wonhee Cho, Yong-Ho Lee, Sun Ha Jee, Justin Y. Jeon

**Affiliations:** 1 Department of Sports Industry, Yonsei University, Seoul, Korea; 2 Department of Internal Medicine, Yonsei University College of Medicine, Seoul, Korea; 3 Exercise Medicine Center for Diabetes and Cancer Patients, Institute of Convergence of Science (ICONS), Yousei university, Seoul, Korea; 4 Institute for Health Promotion, Graduate School of Public Health, Yonsei University, Seoul, Korea

**Keywords:** Resting heart rate, Undiagnosed diabetes, Risk score model, Korean National Health and Nutrition Examination Survey

## Abstract

**OBJECTIVES:**

The purpose of this study was (1) to examine whether the addition of resting heart rate (RHR) to the existing undiagnosed diabetes mellitus (UnDM) prediction model would improve predictability, and (2) to develop and validate UnDM prediction models by using only easily assessable variables such as gender, RHR, age, and waist circumference (WC).

**METHODS:**

Korea National Health and Nutrition Examination Survey (KNHANES) 2010, 2012, 2014, 2016 data were used to develop the model (model building set, n=19,675), while the data from 2011, 2013, 2015, 2017 were used to validate the model (validation set, n=19,917). UnDM was defined as a fasting glucose level ≥126 mg/dL or glycated hemoglobin ≥6.5%; however, doctors have not diagnosed it. Statistical package for the social sciences logistic regression analysis was used to determine the predictors of UnDM.

**RESULTS:**

RHR, age, and WC were associated with UnDM. When RHR was added to the existing model, sensitivity was reduced (86 vs. 73%), specificity was increased (49 vs. 65%), and a higher Youden index (35 vs. 38) was expressed. When only gender, RHR, age, and WC were used in the model, a sensitivity, specificity, and Youden index of 70%, 67%, and 37, respectively, were observed.

**CONCLUSIONS:**

Adding RHR to the existing UnDM prediction model improved specificity and the Youden index. Furthermore, when the prediction model only used gender, RHR, age, and WC, the outcomes were not inferior to those of the existing prediction model.

## INTRODUCTION

According to the Korea National Health and Nutrition Examination Survey (KNHANES), the prevalence of diabetes in individuals over the age of 30 was 13.8%, which is equivalent to 4.94 million cases [1]. Diabetes is the sixth leading cause of mortality in Korea [2]. It is associated with complications such as cardiovascular disease [3], stroke [4], peripheral arterial diseases [5], increased mortality [6] and lowered quality of life [7]. However, the rate of undiagnosed diabetes is 21.4% in the Unite States and 35.0% in Korea, increasing every year [1,8].

Undiagnosed diabetes leads to late treatment initiation, which increases risk of complications [9]. Therefore, early detection of undiagnosed diabetes is an important issue and therefore risk scores for undiagnosed diabetes are being developed worldwide, using the risk factors for diabetes [10-12]. In Korea, Lee et al. [12] developed a model with 80% sensitivity, 53% specificity, 33 Youden index (YI), and area under the curve (AUC) of 0.729 using age, family history, hypertension, waist circumference (WC), smoking, and alcohol consumption from 2001 and 2005 KNHANES data.

Although cardiorespiratory fitness is related to risk factors for diabetes and is an important index for preventing and predicting diabetes [13,14], it has not been used in most prediction models for the risk of diabetes due to the associated difficulty and cost of its measurement. In contrast, resting heart rate (RHR) can be easily measured by anyone without any special equipment and is highly associated with obesity [15], stress [16], sympathetic nervous system [17], insulin [18], maximal oxygen comsumption [19,20], physical strength [21], and levels of physical activity [22]. Thus, RHR is a variable that could be used in risk prediction models for undiagnosed diabetes. In fact, a number of studies have demonstrated that high RHR is associated with the prevalence [23-28] and incidence of diabetes [29-31].

Therefore, this study aimed to (1) determine whether the addition of RHR to an existing undiagnosed diabetes prediction model would improve predictability and validity, and (2) develop an undiagnosed diabetes prediction model using only RHR, age, and WC, and validates the predictability.

## MATERIALS AND METHODS

### Study participants

**Figure 1.**
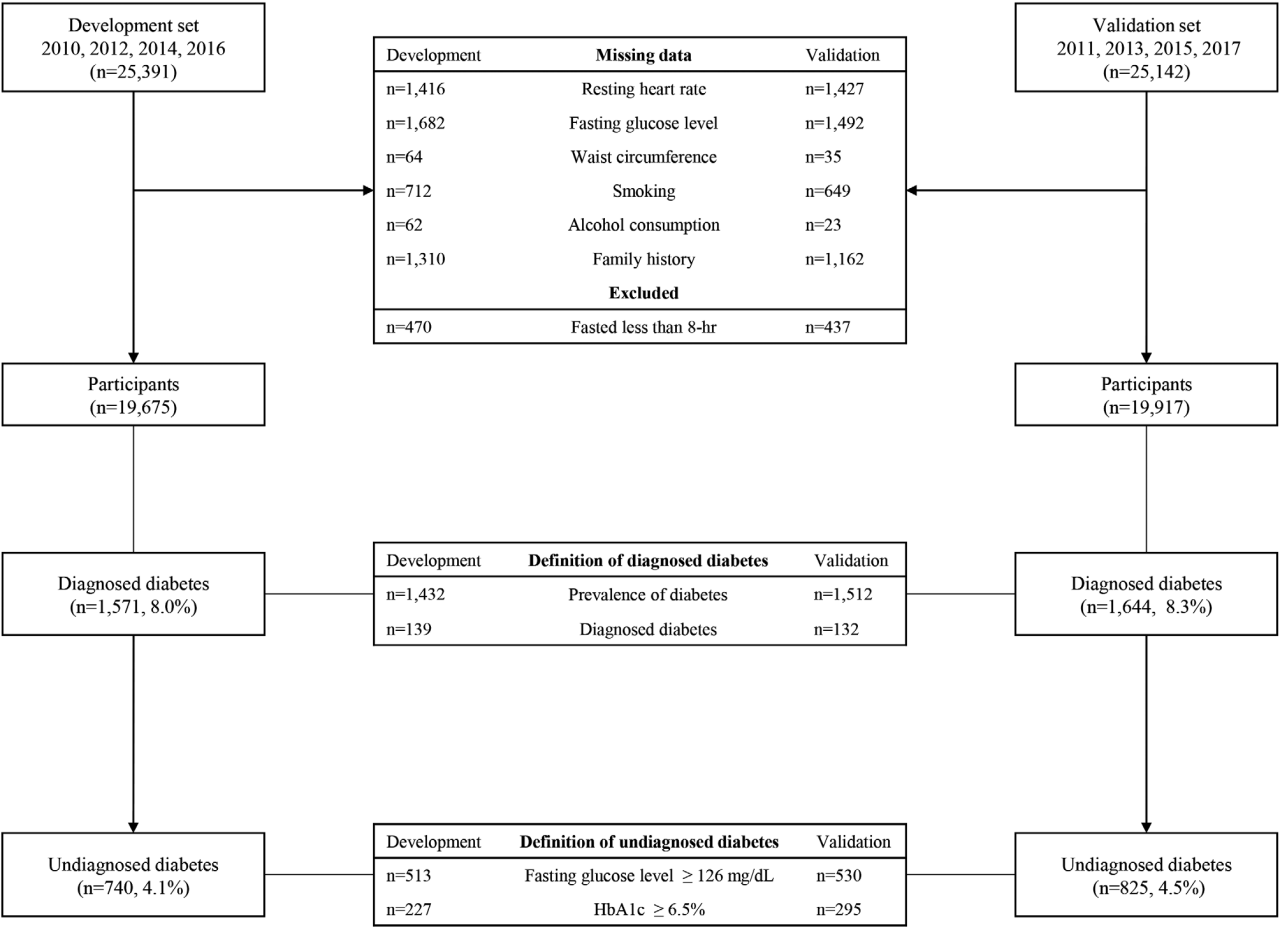
Flowchart for study participants. HbA1c, hemoglobin A1c.

Data from 2010 to 2017 were used in this study [32]. A total of 40,059 participants over the age of 20, after excluding 5,769 with a missing family history of diabetes, 4,646 with missing smoking status, 4,699 with missing data for alcohol intake, 2,817 with missing WC, 5,931 with missing fasting blood glucose level, 2,606 with a missing diagnosis of diabetes, 1,936 who fasted less than 8 hours before blood sample collection, 2,889 with missing values for 60-second RHR, and one participant with RHR of 212 bpm, were analyzed. Data from 19,675 adults from 2010, 2012, 2014, and 2016 KNHANES were used to develop the model, and data from 19,917 adults from 2011, 2013, 2015, and 2017 KNHANES were used to validate the model (Figure 1). The general characteristics of the study participants are listed in Table 1.

### Measurement items and methods

#### Body composition test

Height and weight were measured using a height meter (Seca 225; Seca, Hamburg, Germany) and scale (GL-6000-20; G-TECH, Seoul, Korea), respectively. WC was measured to one decimal place (0.1 cm) using a tape measure (Seca 200, Seca). The center of the lower end of the last rib and the upper end of the iliac crest on the participants’ side were measured [33].

#### Resting heart rate

The RHR was measured as follows: The right arm radial pulse was measured for 15 seconds after completing a questionnaire in a sitting position and resting for more than 30 minutes. If the pulse was irregular, that is, bradycardia (less than 15 beats) or tachycardia (more than 26 beats), the pulse was measured for 60 seconds to assess the regularity of the pulse. The pulse rate for 60 seconds and the number of pulses for 15 seconds converted to 60 seconds were summed and analyzed [33].

#### Undiagnosed diabetes

Participants with fasting blood glucose level greater than 126 mg/dL or glycated hemoglobin (HbA1c) greater than 6.5% (Figure 1) [34], but, have not been diagnosed with diabetes, or under no treatment for diabetes were considered to have undiagnosed diabetes.

#### Previously developed Korean undiagnosed diabetes risk prediction model

Lee et al. [12] developed a Korean undiagnosed diabetes risk prediction model based on age, family history of diabetes, hypertension, WC, smoking, and alcohol intake. The risk index of the model ranged from zero to 11.

#### New Korean undiagnosed diabetes risk prediction model with addition of resting heart rate

The RHR score added to the previously developed undiagnosed diabetes risk prediction model [12] consisted of odds ratio calculated by binary logistic regression analysis of RHR and undiagnosed diabetes. The RHR score was evaluated as follows: zero point for RHR < 60 bpm for men and < 65 bpm for women, one point for RHR of 60-89 bpm for men and 65-84 bpm for women, and two points for RHR of > 90 bpm for men and > 85 bpm for women. The risk index of the model ranged from zero to 13. Moreover, the model consisting of only three variables (RHR, age, and WC) had odds ratio calculated by binary logistic regression analysis of age, WC, and undiagnosed diabetes from KNHANES data of evennumbered years. Age < 40 years, 40-59 years, and > 60 years were given zero, four, and six points, respectively. The WC score was evaluated as follows: zero point for WC of < 84 cm for men and < 77 cm for women, two points for WC of 84-89 cm for men and 77-84 cm for women, and five points for WC of > 90 cm for men and > 85 cm for women. The risk index of the model ranged from zero to 13.

#### Predictive diagnosis criteria

Perkins & Schisterman [35] reported the optimized predictive diagnostic criterion using AUC as the minimum score of (1-sensitivity)2 +(1-specificity)2 or maximum score of YI (sensitivity+ specificity-1) [36]. The predictive diagnostic criteria for each model were: five points for the model by Lee et al. [12], seven points for total, women, and men for the model of current study, and seven points for total, seven points for men, and nine points for women for the model consisting of RHR, age, and WC.

### Statistical analysis

Data were analyzed using the Statistical Package for the Social Sciences version 25.0 statistical program. Frequency analysis, crossanalysis, and independent sample t-tests were conducted for differences in demographic characteristics between men and women elderly individuals. Logistic regression analysis was conducted to estimate the prevalence of type 2 diabetes in the groups according to their RHR [37]. The AUC was also analyzed, and the validity of the model was compared by evaluating sensitivity, specificity, positive predictive value, negative predictive value, positive likelihood ratio, negative likelihood ratio [38], YI (sensitivity+ specificity-1), and AUC [39]. Statistical significance was set at pvalue < 0.05.

### Ethics statement

All procedures including study participants were approved by the Institutional Review Board of the Korea Centers for Disease Control and Prevention (IRB No. 2010-02CON-21-C, 2011-02CON-06-C, 2012-01EXP-01-2C, 2013-07CON-03-4C, 2013-12EXP-03-5C, 2015-01-02-6C, 2018-01-03-P-A).

## RESULTS

### Participants’ characteristics

Participants’ characteristics according to gender are presented in Table 1. Compared to healthy participants (men: 68.40± 0.13 bpm; women: 70.40± 0.11 bpm), participants with undiagnosed diabetes (men: 71.36± 0.48 bpm; women: 71.63± 0.49 bpm) had significantly higher RHR (p < 0.001) and showed significantly higher body mass index, WC, blood glucose level, proportion of family history of diabetes, hypertension, daily alcohol intake, and lower income level.

### Association of resting heart rate, waist circumference and age with prevalence of undiagnosed diabetes

Association of RHR, WC and age with prevalence of undiagnosed diabetes are presented in Table 2. The prevalence of undiagnosed diabetes was approximately 2.14-fold (95% confidence interval [CI], 1.53 to 2.97) higher in the group with the highest RHR than in the lowest RHR group. Additionally, the prevalence of undiagnosed diabetes was 6.68 (95% CI, 5.17 to 8.64) times higher in those over the age of 60 than in those under 40. The prevalence of undiagnosed diabetes was also 5.14 (95% CI, 4.26 to 6.21) times higher in the group with the highest WC than in the group with the lowest WC.

### Validity of the developed Korean undiagnosed diabetes risk prediction model

Validity of the developed Korean undiagnosed diabetes risk prediction model are presented in Table 3. The Korean undiagnosed diabetes risk prediction model, which was previously developed using the results of the 2001 and 2005 KNHANES, was applied to the KNHANES data from even-numbered years (2010, 2012, 2014, and 2016). The sensitivity was 81% for men and 91% for women, and the specificity was 50% and 47% for men and women, respectively. ACU was 0.713 (95% CI, 0.689 to 0.736) and 0.773 (95% CI, 0.752 to 0.794) for men and women, respectively. The YI was 31 for men and 38 for women.

The model was also applied to the KNHANES data from odd-numbered years. The sensitivity was 80% for men and 92% for women, and the specificity was 49% and 47% for men and women, respectively. AUC 0.703 (95% CI, 0.681 to 0.726) and 0.783 (95% CI, 0.763 to 0.802) for men and women, respectively. The YI was 29 for men and 39 for women.

### Validity of Korean undiagnosed diabetes risk prediction model with resting heart rate

Validity of Korean undiagnosed diabetes risk prediction model with RHR are presented in Table 3. The RHR was added to the previously developed undiagnosed diabetes risk prediction model. The model was developed using data from the KNHANES in even-numbered years and validated using data from the KNHANES in odd-numbered years. The sensitivity was 66% for men and 82% for women, and the specificity was 65% and 64% for men and women, respectively. AUC was 0.711 (95% CI, 0.689 to 0.733) and 0.785 (95% CI, 0.766 to 0.804) for men and women, respectively. The YI was 31 for men and 46 for women. Compared to the previous model developed by Lee et al. [12], this new model with RHR had a 14% and 10% decrease in sensitivity for men and women, respectively. The specificity increased by 15% for men and 17% for women, and YI increased by two points for men and seven points for women.

### Validity of Korean undiagnosed diabetes risk prediction model consisting of resting heart rate, age, and waist circumference

Validity of Korean undiagnosed diabetes risk prediction model consisting of RHR, age, and WC are presented in Table 3. A new undiagnosed diabetes risk prediction model was developed using RHR, age, and WC from KNHANES data in even-numbered years and validated using data from KNHANES in odd-numbered years. The sensitivity was 66% for men and 77% for women, and the specificity was 64% for men and 69% for women. AUC was 0.705 (95% CI, 0.683 to 0.727) and 0.786 (95% CI, 0.767 to 0.805) for men and women, respectively. YI was 30 points for men and 46 points for women. Compared to the previous model developed by Lee et al. [12], this new model consisting of only RHR, age, and WC had 14% and 15% decreases in sensitivity for men and women, respectively. The specificity increased by 15% for men and 22% for women, and YI was increased by one point for men and seven points for women.

## DISCUSSION

The main aim of the current study was to investigate whether adding RHR to the Korean diabetes risk prediction model developed in 2012 would affect the model’s predictability. Compared to the previous model [12], the specificity, YI, and AUC increased with the addition of RHR although it is not significant. Knowing that RHR is significantly associated with prevalence of undiagnosed diabetes, lack of significant improvement in prediction model when RHR was added is not because RHR is not an important factor. Rather, RHR was associated with variables used in the previous prediction model such as smoking [40], alcohol intake [41], and hypertension [42] and therefore RHR acts as a mediating or confounding factor in the undiagnosed diabetes risk model. One important observation worth reporting is that addition of RHR to existing prediction model reduced sensitivity but improved specificity of the model, in turn, YI or AUC is similar. When validation analyses were performed, Lee’s models [12] sensitivity was 86% and specificity was 49% while RHR was added to Lee’s model [12], sensitivity decreased to 74% but specificity increased to 65%. Since YI are similar between two model, we cannot say one is better than the other.

The second aim of the current study was to develop and test model using only simple and easily accessible variabes including RHR, age, and WC would predict undiagnosed diabetes. Our results showed prediction model only used RHR were not inferior to previous developed model which used age, family history of diabetes, hypertentioin, WC, smoking, alcohol intake. This results further support discussion that RHR reflects one’s lifestyle factors such as such as smoking [40], alcohol intake [41], and hypertension [42] as previously mentioned. Therefore, the prediction model only used RHR, age, WC perform similar to Lee’s model [12] with RHR added.

Furthermore, our data also showed participants with undiagnosed diabetes were younger than those diagnosed with diabetes, and men with undiagnosed diabetes had significantly higher fasting glucose levels than men with diagnosed diabetes. Taken together with previous studies [9] which showed delay in diabetes diagnosis resulted in late initation of treatment and increased risk of diabetes complication, our findings suggest the importance of early detection, initiation of treatment to control diabetes is of importance and potentially showed the usability of RHR in this process.

Herein, a higher RHR was associated with the prevalence of undiagnosed diabetes. The group with the highest RHR was 2.14 times more likely to have undiagnosed diabetes than the group with the lowest RHR. Li et al. [43] analyzed the relationship between RHR and undiagnosed diabetes in 16,636 participants between the age of 35-78 and reported that men and women with RHR greater than 80 bpm had 3.66-times and 2.98-times higher prevalence of undiagnosed diabetes, respectively, than those with RHR less than 60 bpm. Althogh there are relatively fewer studies investigated association between RHR and undiagnosed diabetes, there are ample number of studies which reported that RHR is associated with revalence and also incidence of diabetes [23-31], suggesting that high RHR is associated with high blood glucose levels regardless of diagnosis of diabetes. Since RHR is associated with obesity [15], stress [16], sympathetic nervous system [17], insulin [18], maximal oxygen comsumption [19,20], physical strength [21], and levels of physical activity [22] and these factors are associated with insulin resistance and glucose metabolism, strong association between RHR and prevalence of diabetes is not surprising.

Our results showed regardless of using Lee’s model [12], Lee’s model [12] plus RHR or model which only used RHR, age, and WC, model’s predictability was better in women than in men. In diabetes models of other countries, more points were allotted to men because men had higher prevalence of diabetes than women [10,11]. However, Lee’s model did not attribute additional points to men and this might have caused some gender differences in the performance of diabete prediction model. Since, the current study followed the same method as Lee’s model, we observed the similar gender difference as Lee’s model.

Our data also showed awareness of diabetes is lower with younger age (46.0% for men and 56.9% for women in their 40s vs. 85.2% for men and 81.5% for women in their 60s), and only 7.0% of men and 5.6% of women in their 40s actively manage their blood glucose levels [44]. The recent advancements in information technology have allowed the creation of advanced medical devices software by combining wearable devices, artificial intelligence, and biomarkers. With advanced medical devices software, patients may prevent, manage, and treat diseases through digital therapeutics [45]. A randomized clinical study was conducted to verify the effectiveness of digital therapeutics in patients with hypertension [46]. Patienttailored analysis and precise medicine will be provided using data measured individually by the patients in the future. In particular, RHR and WC can be measured without special equipment and evaluated in real-time using wearable devices. Thus, it will help people with high diabetes risk to be aware of their risk of diabetes and encourange them to use medical services to manage their health.

Several limitations must be considered when interpreting this study’s findings. Firstly, the KNHANES used to develop and validate the model was a cross-sectional study. The data may have been sufficient to investigate the prevalence of undiagnosed diabetes; however, it may be inadequate to study incidence of diabetes. Therefore, a diabetes risk prediction model must be developed using the incidence of diabetes from prospective cohort data. Secondly, since the oral glucose tolerance test was not performed for KNHANES, only fasting blood glucose and HbA1c were used for determination of undiagnosed diabetes. This may lead to an underestimation of patients with undiagnosed diabetes. Lastly, RHR may fluctuate depending on smoking, alcohol intake, sleep, and physical condition and therefore, caution must be exercised when single measure of RHR is used for risk prediction models.

In conclusion, RHR is highly correlated with the prevalence of undiagnosed diabetes and could be used to increase the predictability of diabetes risk prediction models. Furthermore, the prediction model developed using only RHR, age and WC, which anyone can easily measure, had similar predictability to the previous diabetes risk prediction model. The results of this study may help develop future strategies or applications for predicting early undiagnosed diabetes.

## Figures and Tables

**Table 1.** Characteristics of participants in development set^1^

Characteristics	Men			p-value	Women			p-value
	Non-diabetes (n=7,176)	Undiagnosed diabetes (n=385)	Diagnosed diabetes (n=767)		Non-diabetes (n=10,188)	Undiagnosed diabetes (n=355)	Diagnosed diabetes (n=804)	
Age (yr)	47.88±0.21	56.73±0.78^2,3^	62.50±0.55^2^	<0.001	47.53±0.18	58.15±0.82^2,3^	64.03±0.54^2^	<0.001
Body mass index (kg/m^2^)	24.11±0.04	25.65±0.17^2,3^	24.70±0.12^2^	<0.001	23.14±0.04	26.45±0.18^2,3^	25.19±0.12^2^	<0.001
Waist circumference (cm)	84.51±0.12	89.43±0.45^2^	88.67±0.32^2^	<0.001	77.68±0.11	87.56±0.50^2,3^	85.66±0.33^2^	<0.001
RHR (bpm)	68.40±0.13	71.36±0.48^2^	70.61±0.34^2^	<0.001	70.40±0.11	71.63±0.49^2^	71.73±0.33^2^	<0.001
Fasting glucose (mg/dL)	95.13±0.27	144.78±0.98^2,3^	138.86±0.69^2^	<0.001	92.16±0.21	138.42±0.96^2^	135.47±0.64^2^	<0.001
HbA1c (%)	5.53±0.01	7.10±0.03^2,3^	7.29±0.02^2^	<0.001	5.51±0.01	7.12±0.03^2^	7.26±0.02^2^	<0.001
Family history of diabetes	1,109 (15.5)	80 (20.8)	209 (27.2)	<0.001	1,781 (17.5)	81 (22.8)	226 (28.1)	<0.001
Hypertension	2,338 (32.6)	230 (59.7)	489 (63.8)	<0.001	2,412 (23.7)	182 (51.3)	549 (68.3)	<0.001
Alcohol intake (drinks/day)				<0.001				<0.001
<1	4,680 (65.2)	220 (57.1)	509 (66.4)		9,360 (91.9)	336 (94.6)	781 (97.1)	
1-5	2,022 (28.2)	115 (29.9)	193 (25.2)		745 (7.3)	17 (4.8)	20 (2.5)	
≥5	474 (6.6)	50 (13.0)	65 (8.5)		83 (0.8)	2 (0.6)	3 (0.4)	
Smoking				<0.001				0.287
Never	1,586 (22.1)	62 (16.1)	99 (12.9)		9,116 (89.5)	326 (91.8)	733 (91.2)	
Past	2,677 (37.3)	177 (46.0)	401 (52.3)		549 (5.4)	13 (3.7)	33 (4.1)	
Current	2,913 (40.6)	146 (37.9)	267 (34.8)		523 (5.1)	16 (4.5)	38 (4.7)	
Education				<0.001				<0.001
Elementary	1,665 (23.2)	107 (27.8)	194 (25.3)		2,361 (23.2)	106 (29.9)	223 (27.7)	
Middle school	1,810 (25.2)	100 (26.0)	191 (24.9)		2,530 (24.8)	90 (25.4)	205 (25.5)	
High school	1,832 (25.5)	92 (23.9)	177 (23.1)		2,593 (25.5)	83 (23.4)	193 (24.0)	
College	1,829 (25.5)	80 (20.8)	200 (26.1)		2,627 (25.8)	74 (20.8)	176 (21.9)	
Income				0.136				0.002
Low	913 (12.7)	81 (21.0)	189 (24.6)		2,245 (22.0)	156 (43.9)	456 (56.7)	
Middle low	684 (9.5)	54 (14.0)	150 (19.6)		966 (9.5)	52 (14.6)	118 (14.7)	
Middle high	2,544 (35.5)	117 (30.4)	231 (30.1)		3,445 (33.8)	96 (27.0)	156 (19.4)	
High	2,895 (40.3)	116 (30.1)	179 (23.3)		3,350 (32.9)	42 (11.8)	55 (6.8)	

Values are presented as mean±standard error or number (%). RHR, resting heart rate; HbA1c, hemoglobin A1c; ANCOVA, analysis of covariance.

1 All variables were tested by ANCOVA or chi-square test; ANCOVA was performed with age as covariates.

2 p-value <0.05 vs. non-diabetes.

3 p-value <0.05 vs. diagnosed diabetes.

**Table 2.** Logistic regression analyses for related factors in undiagnosed diabetes in development set

Variables	n (%)	OR (95% CI)	p-value	Score assigned
RHR, men/women (bpm)				
<60/<65	135 (3.0)	1.00 (reference)		0
60-74/65-74	343 (4.0)	1.32 (1.07, 1.61)	0.008	1
75-89/75-84	210 (5.0)	1.68 (1.35, 2.10)	<0.001	1
≥90/≥85	52 (6.3)	2.14 (1.53, 2.97)	<0.001	2
Age (yr)				
<40	72 (1.2)	1.00 (reference)		0
40-59	319 (4.4)	3.90 (3.01, 5.04)	<0.001	3
≥60	349 (7.3)	6.68 (5.17, 8.64)	<0.001	6
WC, men/women (cm)				
<84/<77	159 (1.8)	1.00 (reference)		0
84-89/77-84	195 (3.9)	2.15 (1.74, 2.66)	<0.001	2
≥90/≥85	386 (8.8)	5.14 (4.26, 6.21)	<0.001	5

OR, odds ratio; CI, confidence interval; RHR, resting heart rate; WC, waist circumference.

**Table 3.** Performance of the new and Korean undiagnosed diabetes screening method in the development and validation datasets

Variables		Gender	Cut-off point	High risk (%)	Sensitivity (%)	Specificity (%)	PPV	NPV	PLR	NLR	Youden index	AUC (95% CI)
Development set												
	Lee^1^	Total	5	53	86	49	0.07	0.99	1.67	0.29	35	0.737 (0.721, 0.753)
		Men	5	51	81	50	0.08	0.98	1.63	0.38	31	0.713 (0.689, 0.736)
		Women	5	54	91	47	0.06	0.99	1.73	0.19	38	0.773 (0.752, 0.794)
	Lee^1^+RHR	Total	7	36	73	65	0.08	0.98	2.09	0.42	38	0.748 (0.732, 0.763)
		Men	7	47	77	55	0.08	0.98	1.72	0.41	32	0.719 (0.696, 0.742)
		Women	7	40	80	62	0.07	0.99	2.10	0.32	42	0.780 (0.760, 0.800)
	Park^2^	Total	7	34	70	67	0.08	0.98	2.14	0.45	37	0.747 (0.732, 0.762)
		Men	7	37	67	65	0.09	0.97	1.92	0.51	32	0.710 (0.687, 0.733)
		Women	7	32	73	69	0.08	0.99	2.36	0.39	42	0.778 (0.758, 0.798)
Validation set												
	Lee^1^	Total	5	54	86	48	0.07	0.99	1.65	0.30	34	0.737 (0.722, 0.752)
		Men	5	53	80	49	0.08	0.98	1.56	0.41	29	0.703 (0.681, 0.726)
		Women	5	54	92	47	0.06	0.99	1.74	0.18	39	0.783 (0.763, 0.802)
	Lee+RHR	Total	7	37	74	65	0.09	0.98	2.09	0.41	39	0.750 (0.731, 0.761)
		Men	7	36	66	65	0.10	0.97	1.91	0.52	31	0.711 (0.689, 0.733)
		Women	7	37	82	64	0.08	0.99	2.30	0.28	46	0.785 (0.766, 0.804)
	Park^2^	Total	7	35	71	67	0.09	0.98	2.14	0.43	38	0.749 (0.734, 0.763)
		Men	7	38	66	64	0.10	0.97	1.84	0.53	30	0.705 (0.683, 0.727)
		Women	7	33	77	69	0.09	0.99	2.46	0.33	46	0.786 (0.767, 0.805)

Cut-off point, highest Youden index; PPV, positive predictive value; NPV, negative predictive value, PLR, positive likelihood ratio; NLR, negative likelihood ratio; AUC, area under the curve; RHR, resting heart rate; WC, waist circumference. Korean undiagnosed diabetes screen score (Lee model [16]): (35 years≤age≤44 years)*2+(age ≥45 years)*3+(family history of diabetes)*1+(hypertension)*1+[men: (84 cm≤WC≤89.9 cm), women: (77 cm≤WC≤83.9 cm)]*2+[men: (WC ≥90 cm), women: (WC ≥84 cm)]*3+(current smoker)*1+(1≤drinks/day≤4.9)*1+(drinks/day ≥5)*2.

1 Lee model+RHR: [men: (60 bpm≤RHR≤74 bpm), women: (65 bpm≤RHR≤74 bpm)]*1+[men: (75 bpm≤RHR≤89 bpm), women: (75 bpm≤RHR≤84 bpm)]*1+[men: (RHR ≥90 bpm), women: (RHR ≥85 bpm)]*2.

2 Park model: (40 years≤age≤59 years)*4+(age ≥60 years)*6+[men: (60 bpm≤RHR≤74 bpm), women: (65 bpm≤RHR≤74 bpm)]*1+[men: (75 bpm≤RHR≤89 bpm), women: (75 bpm≤RHR≤84 bpm)]*1+[men: (RHR ≥90 bpm), women: (RHR ≥85 bpm)]*2+[men: (84 cm≤WC≤89.9 cm), women: (77 cm≤WC≤84 cm)]*2+[men: (WC ≥90 cm), women: (WC ≥85 cm)]*5.

## SUPPLEMENTARY MATERIALS

Korean version is available at https://www.e-epih.org/.
