# Supplementary material for: The predictive value of resting heart rate in identifying undiagnosed diabetes in Korean adults: Korea National Health and Nutrition Examination Survey
Source: Epidemiol Health. 2022 Jan 3;44:e2022009. doi: 10.4178/epih.e2022009 (PMC9117096; doi:10.4178/epih.e2022009)
Supplement: Supplementary file 1 [file epih-44-e2022009-suppl.docx]

**The predictive value of resting heart rate in identifying undiagnosed diabetes in Korean adults: Korea National Health and Nutrition Examination Survey**

Resting heart rate and Undiagnosed diabetes

**Dong-Hyuk Park¹, Wonhee Cho^1^, Yong-ho Lee^3^, Sun Ha Jee^2,4^, Justin Y. Jeon ^1,2^**

¹Department of Sports industry, Yonsei University, Seoul, Korea; ²Exercise Medicine Center for Diabetes and Cancer Patients, ICONS, ^3^Department of Internal Medicine, Yonsei University College of Medicine, Seoul, Republic of Korea, ^4^ Institute for Health Promotion, Graduate School of Public Health, Yonsei University, Seoul

**OBJECTIVE:** The purpose of the current study is to examine 1) whether the addition of resting heart rate (RHR) to existing undiagnosed diabetes (UnDM) prediction model would improve predictability, 2) to develop and validate UnDM prediction model by only using easily assessable variables such as age, gender, RHR and waist circumference (WC).

**METHODS:** Korea National Health and Nutrition Examination Survey (KNHANES) 2010, 2012, 2014, 2016 data were used to develop the model (model building set, N=19,675) and KNHANES data from 2011, 2013, 2015, 2017 were used to validate the model (validation set, N=19,917). UnDM was defined as a fasting glucose level ≥ 126mg/dL or hemoglobin A1c ≥6.5%, yet has not been diagnosed by doctors. SPSS logistic regression analysis was used to determine predictors of UnDM.

**RESULTS:** Age, RHR, waist circumference (WC) were associated with UnDM. When RHR was added to the existing model, sensitivity was reduced (86% vs. 73%) while specificity was increased (49% vs. 65%), expressed as higher Youden index (35 vs. 38). When only age, sex, WC and RHR were used in the model, sensitivity of 70%, and specificity of 67% with Youden index of 37 were observed.

**CONCLUSIONS:** Adding RHR to existing UnDM prediction model improved specificity and Youden index. Furthermore, prediction model only used age, sex, RHR and WC were not inferior to existing prediction model.

**KEY WORLD:** Resting heart rate, Undiagnosed diabetes, Risk score model, Korean National Health and Nutrition Examination Survey.

**INTRODUCTION**

국민건강영양조사에 따르면 2016-2018년 30세 이상 당뇨병 유병률은 13.8%로 약 494만 명으로 추정된다 [1]. 당뇨병은 우리나라 사망원인 6위이며 [2], 심혈관 질환 [3]과 뇌졸중 [4], 말초동맥질환 [5]과 같은 합병증을 초래하여 사망률 [6]을 높이고 삶의 질을 떨어뜨리는 중요한 질환 [7] 이지만, 당뇨병 미진단 비율이 미국 21.4%, 국내 35%로 매년 증가하고 있다 [1, 8].

당뇨병은 치료가 늦어질수록 합병증 위험이 높기 때문에 [9], 여러 나라가 당뇨병 위험 요인들을 이용하여 미진단 당뇨병 위험 예측 모델(risk score)을 개발하고 있다 [10-12]. 우리나라는 Lee 등 [12]이 2001, 2005년 국민건강영양조사 자료를 이용하여, 나이, 가족력, 고혈압 유무, 허리둘레 (Waist circumference, WC), 흡연, 음주량을 이용하여 민감도 80%, 특이도 53%, Youden Index (YI) 33점, AUC 0.729인 모델을 개발하였다.

그러나 당뇨병 위험 요인과 연관성이 있는 심폐 체력은 당뇨병을 예방하고 예측하는 지표로서 중요한 변인이나 [13, 14], 측정의 용이성과 비용문제로 대부분의 당뇨병 위험 예측 모델에 사용되지 않았다. 반면에, 안정시 심박수 (Resting Heart Rate, RHR)는 누구나 특별한 장비 없이도 손쉽게 측정이 가능하며 비만도 [15], 스트레스 [16], 교감신경 [17], 인슐린 [18], 최대산소섭취량 [19, 20], 체력 [21], 고강도 신체활동량 [22]과 높은 상관관계가 있음을 고려할 때에, 미진단 당뇨병 위험 예측 모형에 사용할 수 있는 변인이다. 지금까지 많은 연구에서 높은 RHR은 당뇨병 유병률 [23-28]과 발병 위험과 관련이 있다고 규명하였다 [29-31].

따라서 본 연구의 목적은 1) RHR을 기존에 개발된 한국인 미진단 당뇨병 위험 예측 모델에 추가하였을 때에 예측력과 타당도가 기존 모델을 개선하는지 알아보고, 2) 특별한 장비 혹은 혈액검사결과가 없이도 측정이 가능하며 심폐 체력과 연관성이 있는 RHR과 비만도를 나타내는 WC만을 이용하여 미진단 당뇨병 예측 모형을 개발하고, 기존 모델과 예측력과 타당도를 비교해 보는 것이다.

METERIAL AND METHODS

1. 연구 대상

본 연구는 2010년부터 2017년도까지 시행된 국민건강영양조사 자료를 이용하였다 [32]. 본 연구에서 20세 이상 성인(51,126명)의 자료 중 당뇨병 가족력 결측값 5,769명, 흡연 결측값 4,646명, 알코올 섭취량 결측값 4,699명, WC 결측값 2,817명, 공복혈당 결측값 5,931명, 당뇨병 유무에 대한 결측 값 2,606명, 측정일 전날 8시간 공복 미만인 1,963명, 60초 RHR 결측 2,889명, 212 bpm 1명을 제외하고 총 40,059명을 분석하였다. 짝수 년도 (2010, 2012, 2014, 2016) 성인 19,892명을 대상으로 당뇨병 위험 예측 모델을 개발하였고 홀수 년도 (2011, 2013, 2015, 2017) 성인 20,167명으로 당뇨병 위험 예측 모델 타당도를 분석하였다 (Figure 1). 본 연구에 참여한 대상자의 일반적 특성은 Table 1과 같다.

2. 측정 항목 및 방법

**1) 신체구성검사**

신장과 체중은 신장계 (Seca 225, seca, Germany)와 체중계(GL-6000-20, G-TECH, Korea)를 이용하여 측정하였으

며, WC는 대상자의 측면에서 마지막 늑골 하단 및 장골능선 상단 두 지점의 정중앙을 줄자 (Seca 200, seca, Germany)를 이용해 소수점 한자리(0.1 cm)까지 측정하였다 [33].

**2) RHR**

RHR는 30분 이상 앉아서 설문 응답 및 안정을 취한 후 오른팔 요골 맥박을 15초간 측정하였다. 맥박이 불규칙하거나 서맥(15회 미만) 또는 빈맥(26회 이상)일 경우 60초간 맥박을 측정하여 규칙적인지 여부를 확인하였고 60초 동안 맥박을 측정한 값과 15초 동안 측정한 맥박수를 60초로 변환한 값을 합산하여 분석하였다 [33].

3) 미진단 당뇨병

미진단 당뇨병의 진단기준은 대상자가 의사로부터 당뇨병 진단, 현재 당뇨병 유병 여부, 당뇨병 치료 여부, 혈당강하제 약을 복용한 여부를 묻는 문항에 “예”라고 응답한 자 또는 의사로부터 당뇨병을 진단 받은 시기에 응답한 자를 제외하고 미국당뇨병학회 [34]에서 제시한 공복혈당 126mg/dL 이상일 경우 혹은 당화혈색소 6.5% 이상일 경우로 정의하였다 (Figure1). 검사 당일 공복 시간 8시간 미만인 대상자 1,036명을 제외하였다.

4) 기존에 개발된 한국형 미진단 당뇨병 위험 예측 모델

Lee 등 [12]은 나이, 당뇨병 가족력, 고혈압 유무, WC, 현재 흡연 여부, 알코올 섭취량으로 한국형 미진단 당뇨병 위험 예측 모델을 개발하였다. 개발된 모델의 위험지수 범위는 0점에서 11점이다.

5) RHR가 추가된 한국형 미진단 당뇨병 위험 예측 모델

개발된 미진단 당뇨병 위험 예측 모델 [12]에 추가한 RHR의 점수는 RHR와 미진단 당뇨병을 이분형 로지스틱 회귀분석하여 산출된 오즈비 값으로 구성했다. RHR 가 남자 60bpm 미만, 여자 65bpm 미만은 0점, 남자 60-74bpm, 여자 65-74bpm은 1점, 남자 75-89bpm 여자 75-84bpm: 1점, 남자 90bpm 이상, 여자 85bpm 이상: 2점으로 추가하여 모델의 위험지수 범위는 0점에서 13점이다. 또한, 나이, RHR, WC 3가지 변인으로만 구성된 모델은 국민건강영양조사 짝수 연도의 나이, WC와 미진단 당뇨병을 이분형 로지스틱 회귀분석하여 산출된 오즈비 값으로 구성했다. 나이 40세 미만은 0점, 40-59세는 4점, 60세 이상은 6점이며 WC는 남자 84cm미만, 여자 77cm미만은 0점, 남자 84-89cm, 여자 77-84cm는 2점, 남자 90cm이상, 여자 85cm이상은 5점으로 모델의 위험지수 범위는 0점에서 13점이다.

6) 예측 진단 기준

Perkins and Schisterman 등 [35]은 AUC 면적을 이용한 최적화 된 예측 진단 기준을 (1-민감도)^2^+(1-특이도)^2^의 최솟값이 되는 점수 또는 YI (민감도+특이도-1) [36] 의 최댓값이라고 보고하고 있다. 모델의 예측 진단 기준은 각각 Lee 등 [12]의 모델은 5점, Lee 등의 모델에 RHR를 추가한 모델은 전체 7점, 남자 7점, 여자 7점, RHR, 나이, WC로 구성된 모델은 전체 7점, 남자 7점, 여자 9점으로 제시했다.

3. 자료처리방법

본 연구에서의 자료 분석은 SPSS 25.0 통계 프로그램을 사용하였으며, 통계 분석 내용은 다음과 같다. 남성 노인과 여성 노인의 인구학적 특성 차이를 살펴보기 위하여 빈도 분석, 교차분석, 독립표본 t 검정(t-test)을 실시하였다. RHR 수준에 따른 그룹 간에 제2형 당뇨병 유병률을 추정하기 위하여 로지스틱 회귀분석을 실시하였다 [37]. 또한 곡선 아래의 면적(AUC, area under the curve)도 분석하였다. 모델의 타당도는 민감도, 특이도, 양성 예측도, 음성 예측도, 양성 우도비, 음성 우도비 [38], YI (민감도+특이도-1), AUC 면적 [39]을 계산하여 비교하였다. 통계 수준은 *p*<0.05로 설정하였다.

RESULTS

1. 대상자 특성

대상자의 성별에 따른 특성은 <Table 1>과 같다. 정상인과 비교해 (남자 68.4±9.4 bpm, 여자 70.5±9.4 bpm) 미진단 당뇨병 대상자 (남자 71.4±10.9 bpm, 여자 71.6±9.9 bpm)가 RHR이 유의하게 높았으며 (*p*<0.001), BMI, WC, 혈당, 가족력, 고혈압 유무, 하루 알코올 섭취, 수입 수준에 유의한 차이가 있었다. 또한, 연령과 안정시심박수와의 관계를 살펴본 결과, 남성의 경우 연령이 높아질수록 심박수가 낮아지나, 여성의 경우 70대 이후 심박수가 다시 높아지는 패턴을 보였다 (Supplementary 2)

2. 임상 변수와 미진단 당뇨병과의 연관성

임상 변수와 미진단 당뇨병 유병률 연관성의 결과는 <Table 2>와 같다. RHR가 가장 낮은 그룹보다 가장 높은 그룹의 진단받지 않은 당뇨병 유병률이 2.14배 (95%CI: 1.53-2.97) 더 높았으며, 40세 미만과 비교해 나이가 60세 이상 경우 진단받지 않은 당뇨병 유병률이 6.68배 (95% CI: 5.17-8.64) 높았다. 또한, WC가 가장 낮은 그룹에 비해서 가장 높은 그룹은 진단받지 않은 당뇨병 유병률이 5.14배 (95%CI: 4.26-6.21) 더 높은 것으로 나타났다.

3. 개발된 한국형 미진단 당뇨병 위험 예측 모델의 타당도

기존에 2001과 2005년 국민건강영양조사의 결과를 이용하여 개발된 한국형 미진단 당뇨병 위험 예측 모델을 최근 국민건강영양조사 짝수 연도 (2010, 2012, 2014, 2016) 데이터에 적용해봤을 때, 민감도가 남녀 각각 81%와 91%로 나타났으며, 특이도는 남녀 각각 50% 그리고 47%로 나타났다. 곡선 아래의 면적은 남자 0.713 (95%CI: 0.689-0.736), 여자 0.773 (95%CI: 0.752-0.794), 그리고 YI 는 남자 31, 여자 38로 각각 나타났다.

위 모델을 우리나라 국민건강영양조사 홀수 년도 데이터에 적용해봤을 때, 민감도는 남녀 각각 80% 그리고 92%, 특이도는 남녀 각각 49% 그리고 47%로 나타났다. 곡선 아래의 면적은 남자 0.703 (95%CI: 0.681-0.726), 여자 0.783 (95%CI: 0.763-0.802), 그리고 YI 는 남자 29, 여자 39로 나타났다.

4. RHR가 추가 된 한국형 미진단 당뇨병 위험 예측 모델의 타당도

기존에 개발된 모형에 RHR 변인을 추가하여 미진단 당뇨병 위험예측모형을 짝수 연도에서 개발하고 홀수 연도에서 타당도를 검증한 결과 민감도가 남자 66%, 여자 82%, 특이도는 남자 65%, 여자 64%로 나타났다. 곡선 아래의 면적은 남자 0.711 (95%CI: 0.689-0.733), 여자 0.785 (95%CI: 0.766-0.804), YI 는 남자 31, 여자 46로 나타났다. 기존의 Lee 모델을 이용하여 미진단 당뇨병을 예측하였을 때 보다, RHR를 기존 모델에 추가한 민감도는 남녀에서 각각 14%, 10% 감소하였고, 특이도는 남녀에서 각각 15%, 17% 상승하였다. 그 결과 YI 는 남녀에서 각각 2점, 7점 상승한 것으로 확인되었다.

5. RHR, 나이, 허리 둘레로 구성된 한국형 미진단 당뇨병 위험 예측 모델의 타당도

짝수 연도에서 RHR, 나이 그리고 WC만을 이용하여 미진단 당뇨병 위험 예측 모형을 개발하고 홀수 연도 데이터로 타당도를 검사한 결과, 민감도 남자 66%, 여자 77% 로 나타났으며, 특이도는 남자 64%, 여자 69% 로 나타났다. 그리고, 곡선 아래 면적은 남자 0.705 (95%CI: 0.683-0.727), 여자 0.786 (95%CI: 0.767-0.805), YI 는 남자 30점, 여자 46점으로 나타났다. 기존의 Lee모델을 이용하여 미진단 당뇨병을 예측하였을 때 보다, 나이, RHR, WC를 이용한 모델의 민감도는 남녀에서 각각 14%. 15% 감소하였고, 특이도는 남녀에서 각각 15%, 22% 상승하였다. 그 결과 YI 는 남녀에서 각각 1점, 7점 상승한 것으로 확인되었다.

**논의**

본 연구의 첫번째 목표는 2012년 발표한 한국형 당뇨병 위험 예측 모델에 RHR를 추가하여 진단받지 않은 당뇨병 위험 예측도에 영향을 미치는지 알아보는 것이다. 그 결과, 기존의 모델 [12]에 안정시 심박수가 추가된 모델은 기존 모델 보다 특이도, YI, AUC가 높아졌지만 유의하지 않았다. RHR이 당뇨병 유병률과 더 나아가 발생률과도 연관성이 있다는 것이 알려져 있지만, RHR이 추가된 모델이 기존 모델의 당뇨병 예측력을 유의하게 개선하지 못했다는 본 연구의 결과가 RHR이 미진단 당뇨병 위험 예측 모델에 중요하지 않다는 것이 아니다. RHR은 기존 모델에 포함되었던 흡연 [40], 알코올 섭취량 [41], 고혈압 [42]과 상관성이 높음으로 RHR이 위험 예측 모델에서 매개 혹은 혼란 변인의 역할을 하는 것으로 사료된다. RHR이 추가되지 않은 기존 모델과 비교해, RHR이 추가된 위험 예측 모델은 민감도는 85% 에서 74%로 감소하였고, 특이도는 49% 에서 65%로 증가하였다. 그리고, 그 결과 두 모델의 YI와 AUC는 유사하였다. 비록 민감도가 낮아지고 특이도는 증가하였지만, 결국 두 모델의 YI 유사하게 나왔기에, 어느 모델이 더 낫다고 결론 내릴 수는 없다.

본 연구의 두번째 목표는 측정이 간편하고 쉬운 RHR, 나이, WC만 사용하여 만든 미진단 당뇨병을 예측하는 것이다. 본 연구는 RHR, 나이, 그리고 WC 만 사용하여 예측모형을 만들고, 그 타당도를 조사한 결과, 기존에 나이, 가족력, 고혈압, 허리둘레, 흡연 및 음주력을 이용하여 개발한 당뇨 예측 모형과 비교해 당뇨병 유병률 예측력이 열등하지 않다는 것을 확인하였다. 이는 앞에서도 언급했듯이 RHR이 흡연, 알코올 섭취량, 고혈압 유무와 같은 생활습관 요인을 반영하고 있으며, RHR, 나이, 그리고 WC를 이용한 모델이 RHR이 추가된 기존 모델과 유사한 예측력을 가지고 있다는 것을 시사한다.

본 연구에 포함된 미진단 당뇨병 대상자는 당뇨병 환자보다 더 어렸으며, 특히 남성의 경우 당뇨병 남자 환자보다 높은 공복 혈당을 나타냈다. 선행 연구에 따르면 당뇨병 진단이 늦어지면 혈당 조절 비율이 낮고 당뇨병 합병증 위험 증가한다고 보고하였다 [9]. 이에 본 연구의 결과는 당뇨병 조기 발견의 중요성을 시사하며, 이를 위해 RHR이 잠재적으로 유용하다는 것을 나타냈다. 본 연구는 RHR이 높은 그룹이 낮은 RHR 그룹보다 미진단 당뇨병이 2.14배 높은 것을 관찰하였다. Li 등 [43]은 35-78세 16,636명을 대상으로 RHR과 미진단 당뇨병과의 관계를 분석한 결과, RHR이 60bpm 미만인 그룹보다 80bpm 이상인 그룹은 남자 3.66배, 여자 2.98배 미진단 당뇨병 유병률이 높은 것을 보고하였다. 비록 RHR과 미진단 당뇨병 유병률의 연관성 논문은 상대적으로 부족하지만, RHR이 당뇨병 유병률 및 발생률과 관련성이 높다는 사실은 선행연구를 통해 여러 차례 규명되었다 [23-31]. RHR은 비만 [15], 스트레스 [16], 교감 신경계 [17], 인슐린 [18], 최대산소섭취량 [19, 20], 체력 [21], 신체활동량 [22]과 관련이 있고 이러한 요소들은 인슐린 저항성과 포도당 대사와 관련이 있기 때문에 RHR과 당뇨병 유병률 사이의 강한 연관성은 놀랄 일이 아니다.

본 연구에서 개발한 당뇨병 예측 모델의 타당도를 조사한 결과 남자보다는 여자에서 설명력이 높은 것으로 나타났다. 이는 다른 나라 모델은 남자가 여자보다 당뇨병 연관성이 더 높은 것을 고려하여 남자에게 점수를 더 부여 하였지만 [10, 11] 본 연구에서 사용한 Lee 등의 모델은 남자에게 추가 점수를 부여하지 않은 것이 성별로 예측 타당도와 설명력에 차이가 난 것으로 사료된다.

당뇨병 인지율은 나이가 어릴수록 낮고 (40대 남자 46.0%, 여자 56.9% vs. 60대 남자 85.2%, 여자81.5%), 당뇨병 환자의 혈당 조절률은 40대 남자 7%, 여자 5.6%로 대안이 필요한 상황이다 [44]. 최근 IT기술의 발달로 웨어러블 디바이스 , 인공지능 , 바이오마커를 결합하여 고도화된 의료기기 소프트웨어를 만들 수 있게 되었다. 환자들은 고도화된 의료기기 소프트웨어로 근거 기반의 치료 중재를 받음으로써 질병의 예방, 관리, 치료가 가능해졌고 이를 디지털 치료제라고 불리고 있다 [45]. 이미 고혈압 환자를 대상으로 디지털 치료제 효과 검증을 위한 무작위 배정 임상 연구까지 진행되어 [46] 앞으로 환자가 스스로 측정한 데이터로 임상 현장에서 환자 맞춤형 분석과 정밀 의료 서비스 제공이 가능해질 전망으로 보인다. 이에 미진단 당뇨병 위험 모델의 적용 제한점인 환자와 의사의 인식이 디지털 치료제를 통해 변화된다면 손쉽게 당뇨병을 예방하고 치료하는 데 큰 도움이 될 것으로 사료된다. 특히, RHR과 WC는 특별한 장비가 없어도 측정이 가능한 변인이며 웨어러블 디바이스를 이용하면 실시간 관찰이 가능한 변인이기 때문에 미진단 당뇨병 환자들이 침습성 혈당 검사를 받을 수 있도록 도와줄 것이다.

본 연구의 제한점은 다음과 같다. 첫째, 모델의 개발과 타당도 사용된 국민건강영양조사는 단면연구로서 유병률을 알기에 적합하지만 당뇨병 발생을 예측하는 연구로는 부족하다. 따라서 전향적 코호트 데이터를 통해 당뇨병 발생률을 가지고 당뇨병 위험 모델을 만드는 것이 필요하다고 사료된다. 둘째, 본 연구에 사용한 국민건강영양조사 데이터는 경구당부하검사를 실시하지 않았으므로 미진단 당뇨병 대상자는 경구당부하검사를 제외한 공복혈당 126mg/dL 이상과 당화혈색소 6.5%이상인 대상자만 포함 시켰다. 이는 미진단 당뇨병 환자에 대해 과소평가가 될 수 있다는 제한점이 있다. 셋째, 안정시 심박수는 흡연, 음주, 수면, 신체컨디션에 따라 변동성이 있으므로, 당뇨예측모형에 안정시심박수를 포함할 경우 주의가 요구된다.

**결론**

RHR 은 미진단 당뇨병 유병률과 높은 관계를 가지지만, 기존의 당뇨 예측 모형에 안정시심박수를 추가한 경우 예측력을 유의하게 높이지는 못했다. 더 나아가, 누구나 손쉽게 측정할 수 있는 WC와 RHR 그리고 나이를 이용하여 개발한 당뇨병 예측 모형 역시, 기존의 당뇨 예측 모형과 유사한 당뇨예측력을 가진 것으로 규명되었다. 따라서, 향후 조기 미진단 당뇨병을 예측하는 국가 전략 혹은 어플리케이션 개발에 본 연구의 결과가 유용하게 사용될 것으로 사료된다.

**감사의 말씀**

이 연구는 연세대학교 시그니처 사업에 학술연구비의 지원을 받아 연구되었음.

**Reference**

1.Jung C-H, Son JW, Kang S, Kim WJ, Kim H-S, Kim HS, et al. Diabetes Fact Sheets in Korea, 2020: An Appraisal of Current Status. Diabetes & metabolism journal 2021;45:1-10.

2.Korea S. Mortality in Korean. 2018

3.Shah AD, Langenberg C, Rapsomaniki E, Denaxas S, Pujades-Rodriguez M, Gale CP, et al. Type 2 diabetes and incidence of cardiovascular diseases: a cohort study in 1·9 million people. The Lancet Diabetes & Endocrinology 2015;3:105-113.

4.Ergul A, Kelly-Cobbs A, Abdalla M, C Fagan S. Cerebrovascular complications of diabetes: focus on stroke. Endocrine, Metabolic & Immune Disorders-Drug Targets (Formerly Current Drug Targets-Immune, Endocrine & Metabolic Disorders) 2012;12:148-158.

5.Thiruvoipati T, Kielhorn CE, Armstrong EJ. Peripheral artery disease in patients with diabetes: Epidemiology, mechanisms, and outcomes. World journal of diabetes 2015;6:961.

6.Cusick M, Meleth AD, Agrón E, Fisher MR, Reed GF, Knatterud GL, et al. Associations of mortality and diabetes complications in patients with type 1 and type 2 diabetes: early treatment diabetic retinopathy study report no. 27. Diabetes Care 2005;28:617-625.

7.Young BA, Lin E, Von Korff M, Simon G, Ciechanowski P, Ludman EJ, et al. Diabetes complications severity index and risk of mortality, hospitalization, and healthcare utilization. Am J Manag Care 2008;14:15-23.

8.Control CfD, Prevention. National diabetes statistics report, 2020. Atlanta, GA: Centers for Disease Control and Prevention, US Department of Health and Human Services 2020:12-15.

9.Lee YH, Armstrong EJ, Kim G, Oh J, Kang SM, Lee BW, et al. Undiagnosed diabetes is prevalent in younger adults and associated with a higher risk cardiometabolic profile compared to diagnosed diabetes. Am Heart J 2015;170:760-769 e762.

10.Bang H. Development and validation of a patient self-assessment score for diabetes risk.pdf. Ann Intern Med 2009;151:775–783.

11.Griffin S, Little P, Hales C, Kinmonth A, Wareham N. Diabetes risk score: towards earlier detection of type 2 diabetes in general practice. Diabetes/metabolism research and reviews 2000;16:164-171.

12.Lee YH, Bang H, Kim HC, Kim HM, Park SW, Kim DJ. A simple screening score for diabetes for the Korean population: development, validation, and comparison with other scores. Diabetes Care 2012;35:1723-1730.

13.Carnethon MR, Sternfeld B, Schreiner PJ, Jacobs DR, Lewis CE, Liu K, et al. Association of 20-year changes in cardiorespiratory fitness with incident type 2 diabetes: the coronary artery risk development in young adults (CARDIA) fitness study. Diabetes care 2009;32:1284-1288.

14.Zaccardi F, O'Donovan G, Webb DR, Yates T, Kurl S, Khunti K, et al. Cardiorespiratory fitness and risk of type 2 diabetes mellitus: A 23-year cohort study and a meta-analysis of prospective studies. Atherosclerosis 2015;243:131-137.

15.Zhang SY, Wu JH, Zhou JW, Liang Z, Qiu QY, Xu T, et al. Overweight, resting heart rate and prediabetes/diabetes: A population-based prospective cohort study among Inner Mongolians in China. Scientific Reports 2016;6:23939.

16.Shalev AY, Sahar T, Freedman S, Peri T, Glick N, Brandes D, et al. A Prospective Study of Heart Rate Response Following Trauma and the Subsequent Development of Posttraumatic Stress Disorder. Archives of General Psychiatry 1998;55:553.

17.Grassi G, Vailati S, Bertinieri G, Seravalle G, Stella ML, Dell'Oro R, et al. Heart rate as marker of sympathetic activity. Journal of hypertension 1998;16:1635-1639.

18.Anderson EA, Hoffman RP, Balon TW, Sinkey CA, Mark AL. Hyperinsulinemia produces both sympathetic neural activation and vasodilation in normal humans. Journal of Clinical Investigation 1991;87:2246-2252.

19.Gonzales TI, Jeon JY, Lindsay T, Westgate K, Perez-Pozuelo I, Hollidge S, et al. Resting heart rate as a biomarker for tracking change in cardiorespiratory fitness of UK adults: The Fenland Study. medRxiv 2020

20.Nauman J, Aspenes ST, Nilsen TI, Vatten LJ, Wisloff U. A prospective population study of resting heart rate and peak oxygen uptake (the HUNT Study, Norway). PLoS One 2012;7:e45021.

21.Silva DAS, Lima TRd, Tremblay MS. Association between resting heart rate and health-related physical fitness in Brazilian adolescents. BioMed research international 2018;2018

22.Emaus A, Degerstrom J, Wilsgaard T, Hansen BH, Dieli-Conwright CM, Furberg AS, et al. Does a variation in self-reported physical activity reflect variation in objectively measured physical activity, resting heart rate, and physical fitness? Results from the Tromso study. Scandinavian Journal of Public Health 2010;38:105-118.

23.Yang HI, Kim HC, Jeon JY. The association of resting heart rate with diabetes, hypertension, and metabolic syndrome in the Korean adult population: The fifth Korea National Health and Nutrition Examination Survey. Clinica Chimica Acta 2016;455:195-200.

24.Kim D-I, Yang HI, Park J-H, Lee MK, Kang D-W, Chae JS, et al. The association between resting heart rate and type 2 diabetes and hypertension in Korean adults. Heart 2016;102:1757-1762.

25.Park H, Lee J, Kim JY, Kim D-I, Jeon JY. The Relationship between resting heart rate and prevalence of metabolic syndrome and type 2 diabetes mellitus in Korean adults: The fifth Korea National Health and Nutrition Examination Survey (2012). The Korean Journal of Obesity 2015;24:166-174.

26.Cho W, Kim D-i, Min J-h, Jeon JY. The Association of Resting Heart Rate and Muscular Endurance and Prevalence With Type 2 Diabetes in Korean Adults. Exercise Science 2017;26:259-266.

27.Hong JW, Noh JH, Kim D-J. The Association of Resting Heart Rate with the Presence of Diabetes in Korean Adults: The 2010-2013 Korea National Health and Nutrition Examination Survey. PLOS ONE 2016;11:e0168527.

28.Park DH, Hong SH, Cho W, Jeon JY. Higher resting heart rate and lower relative grip strength is associated with increased risk of diabetes in Korean elderly population: Korean national health and nutrition examination survey 2015-2018. Exercise Science 2020;29:416-426.

29.Lee DH, de Rezende LFM, Hu FB, Jeon JY, Giovannucci EL. Resting heart rate and risk of type 2 diabetes: A prospective cohort study and meta-analysis. Diabetes Metab Res Rev 2019;35:e3095.

30.Kim G, Lee Y-H, Jeon JY, Bang H, Lee B-W, Kang E, et al. Increase in resting heart rate over 2 years predicts incidence of diabetes: a 10-year prospective study. Diabetes & Metabolism 2017;43:25-32.

31.Aune D, ó Hartaigh B, Vatten L. Resting heart rate and the risk of type 2 diabetes: a systematic review and dose–response meta-analysis of cohort studies. Nutrition, Metabolism and Cardiovascular Diseases 2015;25:526-534.

32.book KNHANEg. <https://knhanes.kdca.go.kr/knhanes/sub02/sub02_03.do>. 2018

33.Kweon S, Kim Y, Jang M-j, Kim Y, Kim K, Choi S, et al. Data resource profile: the Korea national health and nutrition examination survey (KNHANES). International journal of epidemiology 2014;43:69-77.

34.Association AD. Classification and Diagnosis of Diabetes: Standards of Medical Care in Diabetes—2019. Diabetes Care 2019;42:S13-S28.

35.Perkins NJ, Schisterman EF. The inconsistency of “optimal” cutpoints obtained using two criteria based on the receiver operating characteristic curve. American journal of epidemiology 2006;163:670-675.

36.Youden WJ. Index for rating diagnostic tests. Cancer 1950;3:32-35.

37.Hosmer Jr DW, Lemeshow S, Sturdivant RX. Applied logistic regression: John Wiley & Sons; 2013

38.Brenner H, Gefeller O. Variation of sensitivity, specificity, likelihood ratios and predictive values with disease prevalence. Statistics in medicine 1997;16:981-991.

39.Bradley AP. The use of the area under the ROC curve in the evaluation of machine learning algorithms. Pattern recognition 1997;30:1145-1159.

40.Linneberg A, Jacobsen RK, Skaaby T, Taylor AE, Fluharty ME, Jeppesen JL, et al. Effect of smoking on blood pressure and resting heart rate: a Mendelian randomization meta-analysis in the CARTA consortium. Circulation: Cardiovascular Genetics 2015;8:832-841.

41.Conrod PJ, Peterson JB, Pihl RO, Mankowski S. Biphasic effects of alcohol on heart rate are influenced by alcoholic family history and rate of alcohol ingestion. Alcoholism: Clinical and Experimental Research 1997;21:140-149.

42.Shi Y, Zhou W, Liu X, Ping Z, Li Y-q, Wang C, et al. Resting heart rate and the risk of hypertension and heart failure: A dose–response meta-analysis of prospective studies. Journal of hypertension 2018;36:995-1004.

43.Li YQ, Sun CQ, Li LL, Wang L, Guo YR, You AG, et al. Resting heart rate as a marker for identifying the risk of undiagnosed type 2 diabetes mellitus: a cross-sectional survey. BMC Public Health 2014;14:1052.

44.Association KD. Diabetes fact sheet in Korea 2016. Seoul: Korean Diabetes Association 2018:p6-11.

45.Kim H-S, Kwon IH, Cha WC. Future and Development Direction of Digital Healthcare. Healthcare Informatics Research 2021;27:95-101.

46.Kario K, Nomura A, Kato A, Harada N, Tanigawa T, So R, et al. Digital therapeutics for essential hypertension using a smartphone application: A randomized, open‐label, multicenter pilot study. The Journal of Clinical Hypertension 2021;23:923-934.
